# Effect of ultrasonic treatment on the oral processing characteristics of Mianning ham

**DOI:** 10.3389/fnut.2024.1396623

**Published:** 2024-08-30

**Authors:** Jiaju He, Wenli Wang, Mai Hao, Yue Huang, Lin CHen

**Affiliations:** Key Laboratory of Meat Processing in Sichuan Province, Chengdu University, Chengdu, China

**Keywords:** ultrasound, Mianning ham, oral processing, flavor, TDS

## Abstract

In this paper, the effect of ultrasonic treatment on the oral processing characteristics of Mianning ham was investigated. A sensory evaluation team of 10 evaluators with food professional background was involved in food mastication and dough collection. Oral processing analysis of ultrasonically treated hams was performed using particle distribution analysis, Headspace Solid-Phase Microextraction Gas Chromatography–Mass Spectrometry (SPME-GC–MS), electronic nose, and dynamic dominant sensory attribute testing. The results showed that compared with the control group, the chewing time and the number of chewing times of the ultrasonically treated hams during oral processing were significantly increased, the salivary content in the ham eating dough was significantly reduced, the types and contents of flavor substances were significantly increased, and the ultrasonic treatment significantly reduced the dominant organoleptic attributes such as saltiness and sourness of the Mianning hams. This paper takes Mianning ham bolus as the research object, analyzes the influence of ultrasonic treatment on the flavor perception of Mianning ham, and provides a theoretical basis for the optimization of ham back-end processing technology.

## 1 Introduction

Dry-cured ham is popular among consumers because of its unique aroma and nutritious characteristics (1). Mianning Ham is one of the famous hams in China, which is made from the hind legs of high-quality Wujin pigs, cured, washed and dried, air-dried and fermented for a long time. Mianning ham has thin skin, tender meat, bright red meat, with a unique curing flavor easy to preserve, the main study of Mianning ham at present focuses on the microflora of the ham itself and the change of flavor, the flavor perception of the ham in different process treatments has not been reported (2, 3).

Ultrasonic technology has been used in food processing such as brining, marinating and cooking (4). The cavitation effect created can shorten the curing time of meat products by accelerating salt diffusion (5). Studies have shown that in addition to reducing the amount of salt used by accelerating the rate of salt diffusion, ultrasonic treatment can also affect other physicochemical properties of the product, such as hardness, water-holding capacity, flavor, and so on (6, 7).

Oral processing of food involves occlusion, mastication, transportation, dough formation and swallowing, which are necessary for food digestion and the perception of food texture and flavor (8). When food enters the mouth, it generally undergoes a variety of physical (chewing and salivary lubrication) and chemical (proteins and enzymes) processes that result in the formation of a bolus that can be swallowed (9). A bolus is a mass of food that is chewed and then lubricated by saliva (10). Oral processing perception of food can be divided into texture perception and flavor perception (11).

Volatile compounds in food dissolve in saliva, evaporate into the air through the air-water balance, and are then perceived by the olfactory organs. Non-volatile compounds dissolved in saliva are detected by the taste organs (12). Tian et al. (13) conducted a flavor perception study on different chewing stages of dry-cured pork, with measurements of salivary flow rate, salivary protein content, salivary pH, conductivity, salivary and sodium salts, as well as taste component analyses. It has been found that interactions between food and saliva during oral processing can lead to a significant increase in the perception of salty and sweet flavors (13). Djekic et al. (14) investigated the relationship between oral processing characteristics and sensory and textural profiles of hams in the oral cavity after three different treatments: steaming, vacuum low-temperature cooking and grilling. The results showed that chewing texture and cooking loss were positively correlated with the number of chews, chewing time and salivary participation. The juicier and more tender the ham, the better the sensation in the mouth (14). The flavor and sensory perception of food can also be assessed by the Temporal dominance of sensations (TDS), which explains dynamic perceptual differences during mastication through bouls variability (15) (see Table 1). Textural sensory attributes such as juiciness, tenderness and toughness are also key attributes in determining ham quality and acceptability (16). At present, the study of texture and flavor of food is mainly on the food itself and the processing technology, and there are fewer studies on the flavor changes in the real oral cavity. Therefore, it is meaningful to study the effect of ultrasonic treatment on the oral processing characteristics of Mianning ham.

The objective of this study was to investigate the effect of ultrasonic treatment on oral processing characteristics such as chewing parameters and flavor of Mianning ham.

## 2 Materials and methods

### 2.1 Ham sample processing

Mianning ham samples were provided by local representative factories in Mianning County with hams fermented and matured for 1 year, and the experiment was divided into four groups. The ham samples were changed to 5*5*2 cm slices of ham, and 1% of ultrapure water was added to each sample before vacuum packaging, and the packaged samples were put into the ultrasonic machine for ultrasonic treatment (17). Samples should be wiped of surface moisture and vacuum packed for immediate use or refrigerated at 4°C until use. Ham samples were cooked in boiling water for 10 min, and after cooking, the hams were cut into cubes weighing 5 ± 1 g each for subsequent oral processing experiments (18). The optimized samples were divided into four treatment groups optimal desalination group (UD), optimal hardness group (UH), desalination control group (CD), and hardness control group (CH).CD (84.56 min, 30.35°C, no ultrasound), UD (84.56 min, 30.35°C, ultrasound power 150.85 W), CH (60.67 min, 35.37°C), no UD (84.56 min, 30.35°C, ultrasound power 150.85 W), CH (60.67 min, 35.37°C, no ultrasound), UH (60.67 min, 35.37°C, ultrasound power 130.08 W).

### 2.2 Oral processing

A team of 10 persons (5 males and 5 females; 22 ± 5 years old) with a professional background in food for sensory evaluation was assembled, and the evaluators were free of abnormalities in the oral cavity and in good health. Sensory training was provided to team members, and evaluators were not allowed to smoke, drink alcohol, or consume heavily flavored beverages 2 h prior to participating in sensory testing (19). The same group of evaluators also participated in experiments such as Temporal dominance of sensations (TDS), Oral Processing Analysis, and Bolus Collection.

In order to study the changing patterns of physicochemical properties and sensory changes of ham during oral processing, ham bolus at 25, 50, 75, and 100% chewing time points were collected for subsequent experiments, in which each evaluator’s normal chewing and swallowing time was recorded as the 100% chewing time point. Test ham bolus immediately after collection, or wipe off surface moisture and store in the refrigerator at 4°C (20).

Chewing parameters (chewing time, number of chews, etc.) of the evaluators were recorded through a video camera, and the evaluators took a 3-min break after each chew and cleaned their mouths with mineral water, repeating the process three times (21). Chewing frequency and feeding rate were calculated according to Equations 1, 2, respectively.


(1) Chewing frequency=number of chews/time spent chewing.

(2) Eating rate=weight of food eaten/chewing time.

### 2.3 Particle size analysis

In order to analyze the degree of chewing at different chewing times, ham bolus were collected from different chewing stages. It was rinsed using distilled water and filtered before being placed on a dry white tray and photographs were taken using a scanner (Canon DR-G2110, United States) under the same conditions of background color. Image analysis was performed using ImageJ software (National Institutes of Health, Version 1.45 K) (22). Calculate particle size distribution, number of particles, and particle area size distribution.

### 2.4 Water content and saliva incorporation

To avoid loss of moisture by evaporation, the moisture content and saliva content of the bolus were determined immediately after collection. Bolus with 25, 50, 75 and 100% swallowing points were selected. A 5 g sample of ham bolus was weighed and placed in a moisture meter to determine the moisture content of the bolus, which was expressed as g/g (23).

Saliva content was determined as shown below: Saliva content = Dough moisture content—Sample moisture content. Saliva flow rate = saliva content * 1000 / number of chews.

### 2.5 Bolus flavor analysis

SPME-GC–MS analysis: Accurately weighed 3.0 g of ham food boluss at different chewing stages into a 15 mL headspace vial, 1 μL of 2,4,6-trimethylpyridine was added to the headspace vial as an internal standard, and the headspace vial was sealed (24). The volatile flavor substances were extracted by headspace solid-phase microextraction (SPME). The gas collection was performed using a CTC autosampler with the following settings: heating chamber temperature of 40°C, extraction time of 60 min, and sample analysis time of 5 min, and the volatile flavor substances were analyzed using gas chromatography–mass spectrometry (GC–MS) (25).

Gas chromatographic conditions: An HP-5 ms-UI column (30*0.25 mm, 0.25 m) was used with a column pressure of 32.0 kpa, a constant flow rate of 1.0 mL/min, helium as the carrier gas without split mode, and an inlet temperature of 250°C. The column temperature was controlled by a program, the starting temperature was 35°C, kept for 20 min, increased to 200°C at a rate of 5°C/min, kept for 1 min, and then increased to 250°C at a rate of 15°C/min and kept for 4 min.

Mass spectrometry conditions: an electron ionization source (EI) was used with an electron energy of 70 e V, an ion temperature of 280°C, a temperature of 150°C for the transmission line, a scanning mass range of 35–500 m/z, a scan rate of 1 scan/s, and a detector voltage of 350 V.

Analysis: Qualitative analysis was performed by comparing the chromatograms of the samples obtained through the NIST database and matching the volatile compounds corresponding to the peaks on the chromatograms, with a match of 80% for the L library. Quantitative analysis is performed by normalizing the peak area of the total ion chromatogram to obtain the relative amount of each component of the sample.

### 2.6 Electronic nose analysis

Electronic nose analysis: 5.0 g of food bolus from different chewing stages were accurately weighed into a 15 mL headspace bottle and sealed. An electronic nose assay was performed using an electronic nose injection tip inserted into a headspace vial containing the sample. Electronic nose measurement conditions: sampling time of 5 s/group, sensor cleaning time of 60 s, sensor zeroing time of 5 s, sample preparation time of 5 s, inlet flow rate of 200 mL/min, and analytical sampling time of 60 s (26).

**Table 1:** Description of the performance of each sensor of the electronic nose.

Sensor Name	Representative compound	Performance description
W1C	Aromatic compound	Sensitive to aromatic ingredients, benzene
W5S	Broader	Sensitive to nitrogen oxides
W3C	Aromatic compound	Sensitive to aromatic ingredients, ammonia
W6S	Hydrogen	Sensitive to hydrogen compounds
W5C	Alkane compounds	Sensitive to alkane aromatics
W1S	Methane	Sensitive to methane
W1W	Sulfide	Sensitive to sulfides
W2S	Alcohol compound	Sensitive to alcohols, aldehydes and ketones
W2W	Organic sulfide	Sensitive to organic sulfides, aromas
W3S	Methane-aliphatic compounds	Sensitive to long chain alkanes

### 2.7 Temporal dominance of sensations (TDS)

TDS is a temporal sensory analysis of food products that measures the strength of the dominance of sensory attributes over time by generating a set of perceptual attributes that are classified as “dominant” at specific points or time periods in a dynamic evaluation process. Prior to the TDS evaluation, evaluators are trained in the appropriate sensory aspects of ham, such as hardness, saltiness, juiciness, gumminess, acidity, and softness (27). The definitions of the above sensory attributes were also explained to the evaluator in accordance with international standards (ISO-5492, 2008).

Evaluation team members felt the dominant sensory attribute when they started chewing the sample. They were able to select the same or different sensory attributes at the same time and stopped feeling the sensory attributes moments before swallowing (28). Each evaluator provided 3 samples at 3 min intervals and was required to rinse his/her mouth with purified water before each evaluation.

### 2.8 Statistical analysis

The physicochemical, textural and sensory data were analyzed by ANOVA using SPSS software with T (Tueky’s) test and the results were considered highly significant at *p* < 0.01, significant at 0.05 > *p* > 0.01 and non-significant at *p* > 0.05. All experiments were replicated three times, and Origin was used for graphing.

## 3 Results and discussion

### 3.1 Oral processing analysis

The food is reduced in structure by the forces exerted by the teeth and tongue, in the process increasing the lubrication of the food due to the admixture of saliva, and finally forming a bolus that can be safely swallowed (29). The results of ham chewing after ultrasound treatment are shown in Table 2.

**Table 2:** Oral processing measurement results.

Group	Oral-exposure time (s)	Number of chews	Chewing rate (chew/s)	Bolus moisture content (g/g)	Saliva content (g/g)	Saliva flow rate (mg/s)
25%CD	7.67 ± 0.74^Cd^	7.4 ± 1.43^Bd^	1.06 ± 0.17^Ba^	48.81 ± 0.96^Cd^	7.12 ± 0.96^Bc^	0.94 ± 0.17^Bb^
50%CD	15.35 ± 1.48^Cc^	14.0 ± 1.25^Cc^	1.10 ± 0.08^Ba^	57.08 ± 0.90^Cc^	15.38 ± 0.90^Ab^	1.01 ± 0.11^Ab^
75%CD	23.02 ± 2.21^Cb^	27.1 ± 2.77^Cb^	0.85 ± 0.02^Ab^	68.83 ± 1.43^Ab^	27.13 ± 1.43^Aa^	1.19 ± 0.10^Aa^
100%CD	30.70 ± 2.95^Ca^	35.0 ± 3.02^Ba^	0.88 ± 0.03^Ab^	70.15 ± 1.72^Aa^	28.45 ± 1.72^Ba^	0.93 ± 0.08^Ab^
25%UD	9.93 ± 1.10^Ad^	8.3 ± 1.25^Ad^	1.21 ± 0.10^Aa^	58.39 ± 1.06^Ad^	10.54 ± 1.06^Ad^	1.07 ± 0.17^Ba^
50%UD	19.85 ± 2.19^Ac^	17.7 ± 1.77^Ac^	1.12 ± 0.10^Ab^	62.93 ± 1.15^Ac^	15.08 ± 1.15^Ac^	0.77 ± 0.15^Bb^
75%UD	29.78 ± 3.29^Ab^	35.1 ± 4.20^Ab^	0.85 ± 0.04^Ac^	69.19 ± 1.17^Ab^	21.33 ± 1.17^Cb^	0.72 ± 0.09^Cb^
100%UD	39.71 ± 4.38^Aa^	44.6 ± 5.72^Aa^	0.89 ± 0.04^Ac^	71.05 ± 1.70^Aa^	23.19 ± 1.70^Da^	0.59 ± 0.07^Cc^
25%CH	7.58 ± 0.64^Cd^	6.5 ± 1.35^Bd^	1.21 ± 0.23^Aa^	43.96 ± 1.37^Dd^	3.21 ± 1.37^Cd^	0.42 ± 0.18^Cd^
50%CH	15.17 ± 1.27^Cc^	14.8 ± 1.87^Bc^	1.04 ± 0.12^Bb^	51.70 ± 2.13^Dc^	10.95 ± 2.13^Bc^	0.73 ± 0.16^Bc^
75%CH	22.75 ± 1.91^Cb^	30.1 ± 3.76^Cb^	0.77 ± 0.10^Bc^	66.71 ± 1.82^Cb^	25.96 ± 1.82^Ab^	1.15 ± 0.14^Aa^
100%CH	30.33 ± 2.55^Ca^	40.8 ± 3.19^Aa^	0.75 ± 0.08^Bc^	70.92 ± 1.49^Aa^	30.18 ± 1.49^Aa^	1.00 ± 0.12^Ab^
25%UH	9.03 ± 0.95^Bd^	7.0 ± 0.82^Bd^	1.29 ± 0.07^Aa^	56.16 ± 1.14^Bd^	10.79 ± 1.14^Ac^	1.21 ± 0.23^Aa^
50%UH	18.06 ± 1.89^Bc^	16.0 ± 1.41^Bc^	1.13 ± 0.07^Ab^	60.64 ± 0.95^Bc^	15.27 ± 0.95^Ab^	0.86 ± 0.12^Bb^
75%UH	27.10 ± 2.84^Bb^	32.3 ± 2.83^Bb^	0.84 ± 0.03^Ac^	68.38 ± 1.02^Ab^	23.01 ± 1.02^Ba^	0.86 ± 0.12^Bb^
100%UH	36.13 ± 3.79^Ba^	40.9 ± 4.12^Aa^	0.88 ± 0.02^Ac^	70.10 ± 1.38^Aa^	24.73 ± 1.38^Ca^	0.69 ± 0.10^Bc^

Values are expressed as mean ± standard deviation; every 4 rows are grouped together, with lowercase letters indicating significant differences (*p* < 0.05) between different stages in the same group, and uppercase letters indicating significant differences (*p* < 0.05) between different groups in the same stage.

As shown in Table 2, the presence or absence of ultrasonic treatment and different ultrasonic treatment conditions had a significant effect on oral processing parameters such as mastication time and number of chews. There was a significant difference in the chewing time and number of chews required to reach the swallowing point in the ultrasonically treated Mianning hams compared to the non-ultrasonicated group (*p* < 0.05). The chewing time and the number of chews required to reach the swallowing point were significantly more (*p* < 0.05) in the ultrasonically treated Mianning hams than in the non-ultrasonically treated ones because the ultrasonication leads to a decrease in the salt content in the Mianning hams and an increase in the chewing time of the evaluators (30). The frequency of mastication varied among the different degrees of mastication in the same group, and was significantly higher in the pre-masticatory period than in the post-masticatory period. This may be due to the fact that salinity is the dominant sense in Mianning ham when the evaluator first starts chewing, and the evaluator chews faster, and salinity is not the dominant sense in the later stages of chewing, so the chewing frequency is slower. There was a significant difference in chewing frequency between the pre-sonicated and unsonicated chewing periods, with the ultrasonic treatment leading to a reduction in salt content, as well as a reduction in the evaluator’s perception of saltiness, leading to a difference in chewing frequency.

Moisture content at the initial stage of mastication varied according to ultrasound, and there was also a significant difference in moisture content between the different ultrasound treatment groups. Juice outflow from the ham matrix after ultrasound treatment resulted in an increase in moisture content, again the higher the ultrasound power and the longer the time, the more juice outflow from the ham matrix, so there was a significant difference between the UD and UH groups (31). Mianning ham increases the moisture content of the bolus during chewing, and at the end of chewing, the moisture content of all the bolus reaches about 70%. Although the initial moisture content varies from ham sample to ham sample, saliva makes up the difference during chewing. This result is consistent with the findings of Loret et al.’s study on the oral processing of breakfast cereals, which differed in their initial moisture content, but their moisture content at the point of swallowing was consistent (32). Some authors have suggested that the release of juices from the meat matrix in ham also leads to changes in moisture content, but this is negligible compared to saliva (33).

Saliva is extremely important in oral processing; taste stimuli must be dissolved in saliva during taste perception in order to reach and interact with receptor targets, and saliva also plays an important role in lubricating food into bolus (34). Ultrasonic treatment and chewing time had a significant effect on saliva content (*p* < 0.05), in the same treatment group saliva content increased with increasing degree of chewing, but the increase was significantly lower in the later stages of chewing, which may be due to the fact that in the later stages of chewing there is less change in the moisture content of the bolus, resulting in lower saliva intake. In the different treatment groups, the amount of saliva ingested was significantly different because of the difference in initial water content, but there was no significant difference in the water content of the bolus at the time of swallowing. Therefore, there was a significant difference in salivary content at the time of swallowing, with the UD group having the highest initial water content and the lowest salivary content at the time of swallowing, and vice versa in the CH group. Salivary flow rates in UD and UH decreased with increasing levels of chewing. Salivary flow rates in the CD and CH groups increased with the degree of chewing. This may be due to the fact that the different water content in the pre-chewing period resulted in different salivary intake in the pre-chewing period, and by the late chewing period the increased salivary intake content in the CD and CH groups resulted in a significantly higher salivary flow rate than that of the UD and UH groups.

### 3.2 Bolus particle size analysis

**Figure 1:**
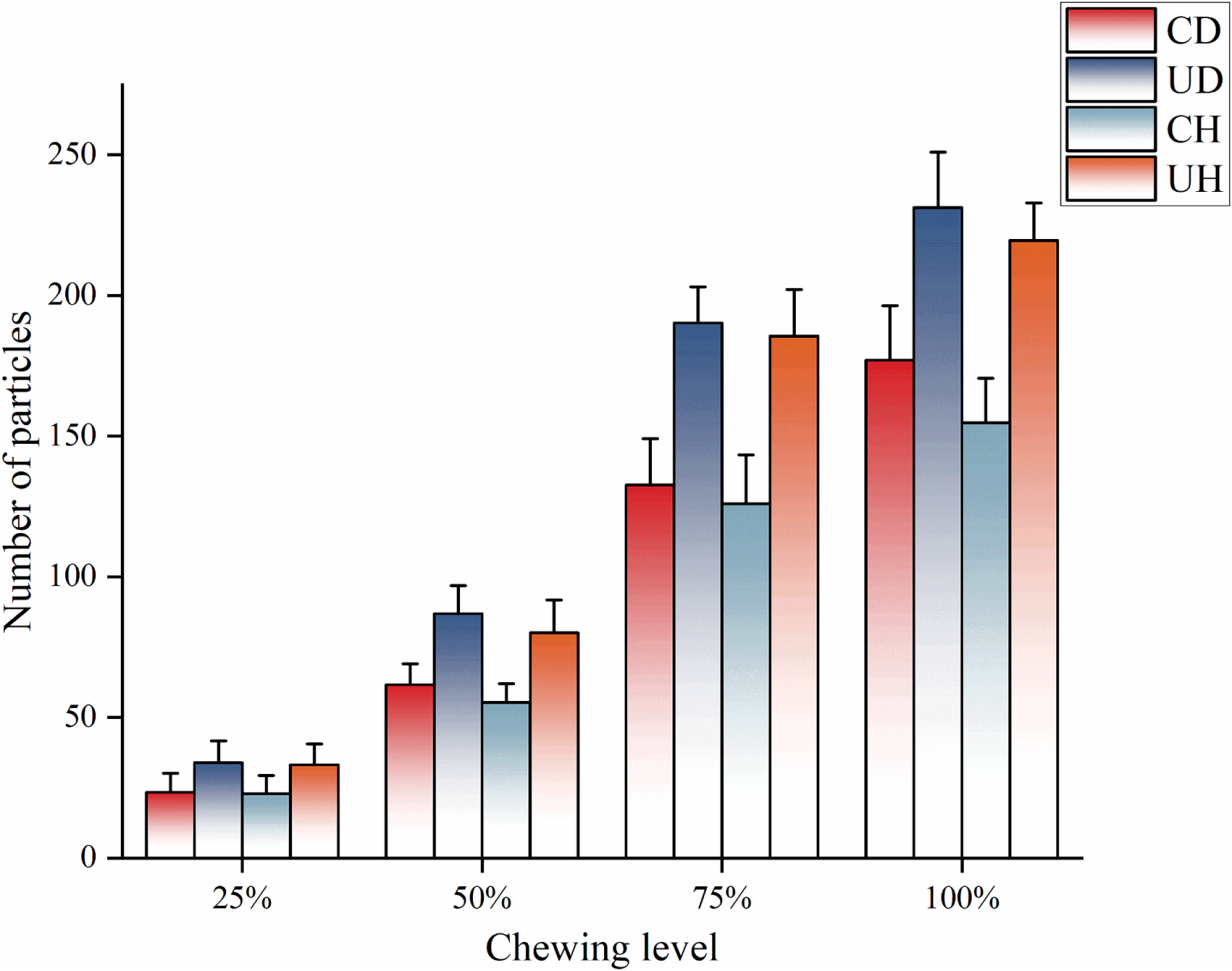
Number of pellet particles for different levels of chewing.

During oral processing, ham is chewed until it becomes a bolus and is swallowed. The smaller and more numerous the bolus, the easier it is to reach the swallowing point (35). Changes in particle size and number were investigated by image analysis of bolus using a scanner. Figure 1 shows the number of bolus particles at 25, 50, 75 and 100% chewing stages for the four groups CD, UD, CH and UH. The UD group had the highest number of particles in all the different levels of chewing, which may be due to the long chewing time of the UD group. The longer the bolus is chewed, the higher the number of bolus particles. Also as the chewing time increases, the number of bolus particles increases. From Figure 1, it can be seen that the number of bolus particles is positively correlated with the chewing time, and the chewing time is UD, UH, CD, and CH in order from longest to shortest, and the number of bolus particles at the same degree of chewing is UD, UH, CD, and CH in order from most to least.

**Figure 2:**
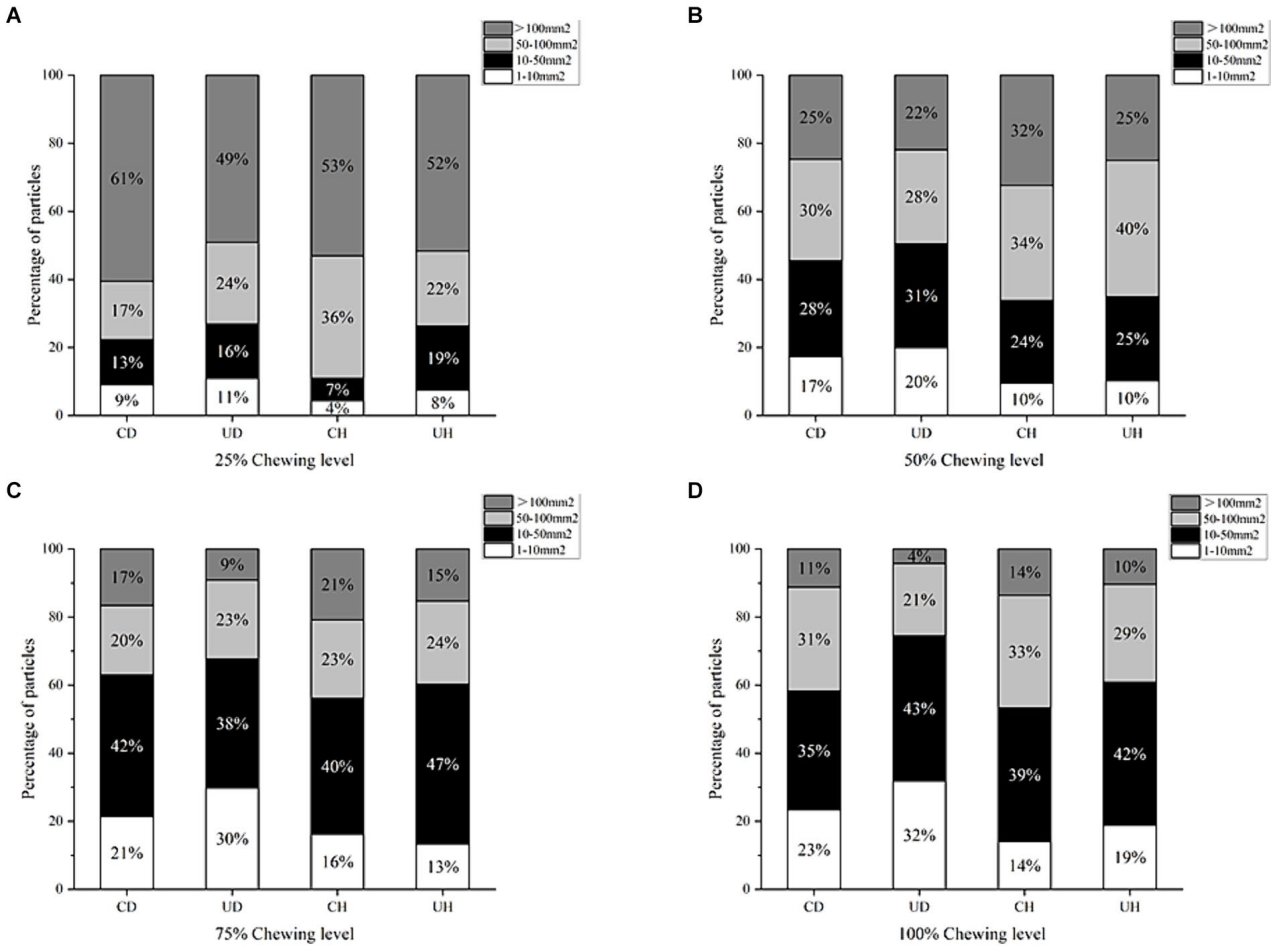
Percentage of particle area in different levels of chewing (**(A)**: 25% Chewing level, **(B)**: 50% Chewing level, **(C)**: 75% Chewing level, **(D)**: 100% Chewing level).

The proportion of particle area in different degrees of chewing is shown in Figure 2. During mastication, the percentage of large particles in the pellet decreased and the percentage of small particles in the bolus increased as the degree of mastication increased. The 25% mastication level was dominated by bolus particles larger than 100 mm^2^, with the highest number of particles in the 10–50 mm^2^ range at the 75 and 100% mastication levels. Changes in the granularity of bolus varied between treatment groups. As shown in Figure 2A the CD group had the highest number of bolus particles larger than 100 mm^2^ at the 25% mastication stage, which may be due to the fact that the CD group had the shortest mastication time resulting in insufficient mastication when the food was formed into a bolus, and a large portion of the bolus was still unchewed. Chewing time in the CH group did not differ much from the CD group but was less hard than the CD group, resulting in the CH group having the fewest particles of 1–10 mm^2^ in 25% of the chewing phase. During mastication, there were more 1–10 mm^2^ bolus particles in the UD than in the other groups, because the UD group chewed for significantly more time than the other treatment groups, and more thorough chewing resulted in smaller bolus particles.

Large particles (>100 mm^2^) were lower in the UD group in the 100% chewing phase, which was related to the depth of chewing. Higher large particles of bolus at 100% chewing degree in the CD and CH groups suggests that these groups are less fragmented in oral processing, the size of bolus particles at the point of swallowing is larger than in the other treatment groups. Differences in particle size of bolus at the point of swallowing have been shown to be related to food hardness through previous studies (36). However, as can be seen in Figure 2, the particle size of the bolus at the point of swallowing is not related to hardness, but to the degree of product fragmentation, which is related to the chewing time.

### 3.3 Flavor analysis

#### 3.3.1 Volatile compounds

**Figure 3:**
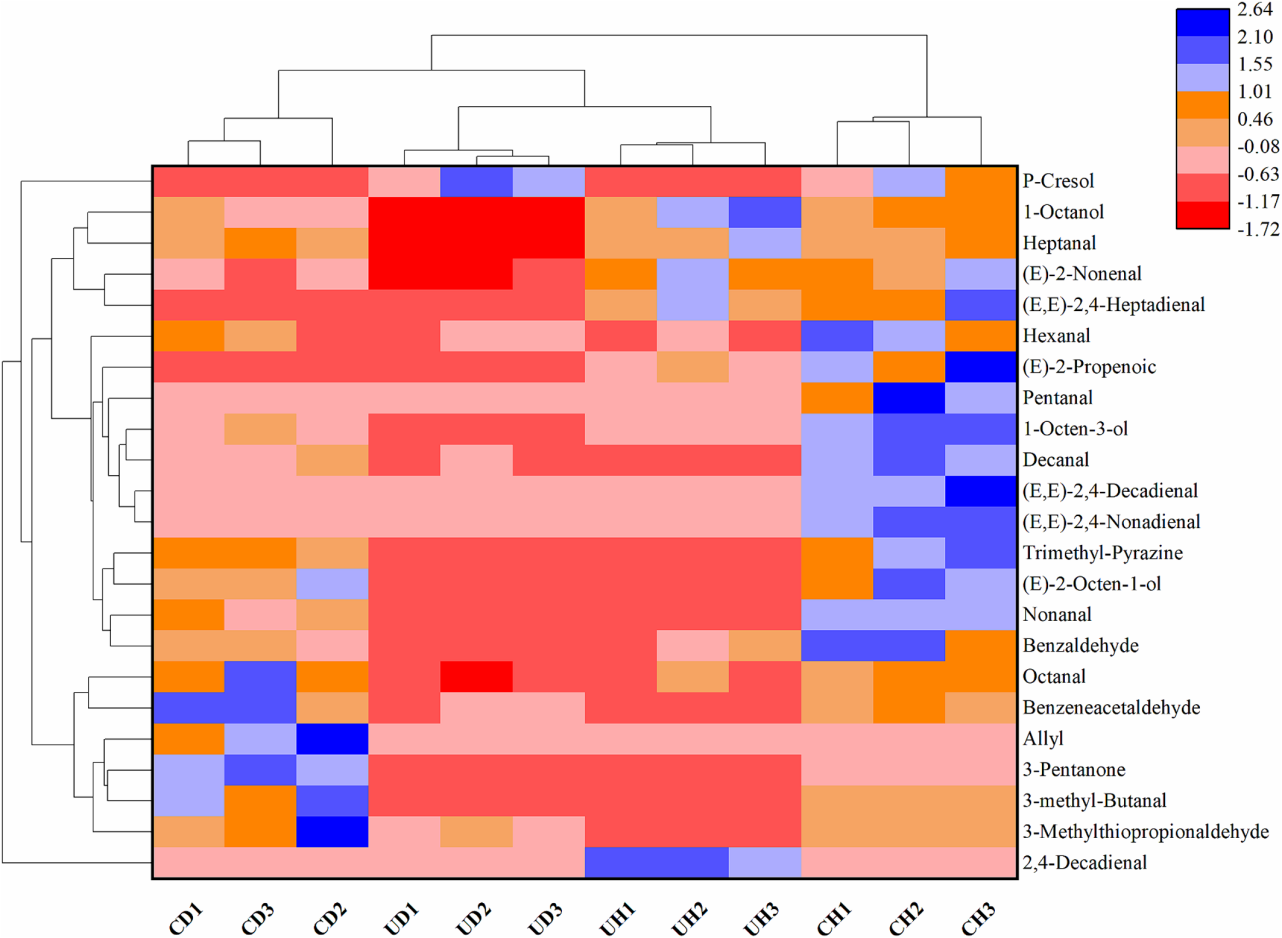
Heat map of clustering of key flavor substances.

In order to understand the key volatiles in different chewing stages of Mianning ham, the food dough in different chewing stages was analyzed by SPME-GC–MS. And referring to the threshold values of the Compendium of Olfactory Threshold Values of Compounds, 23 key volatile flavor substances were screened by the OAV value ≥1 criterion as shown in Table 3. There are 15 aldehydes, 1 ketones, 2 esters, 3 alcohols and 2 others. There was a significant difference (*p* < 0.05) in flavor among the four groups CD, UD, CH, and UH as shown by the clustered heat map of key flavor substances (Figure 3). The key flavor compounds are dominated by aldehydes and alcohols, volatile compounds that may be volatile flavor substances produced by the chemical degradation of esters and amino acids in ham during chewing (37).

**Table 3:** Key volatile flavor substances.

Compound	Thresholds (ug/kg)	OAV value (OAV ≥ 1)			
		CD	CH	UD	UH
2,4-Decadienal	3.50	–	–	–	2.60 ± 0.42
3-Methylthiopropionaldehyde	40.00	5.20 ± 2.80	2.11 ± 0.56	3.10 ± 0.54	–
Benzaldehyde	50.00	8.15 ± 0.80	3.70 ± 0.51	13.07 ± 1.72	5.70 ± 2.15
Benzeneacetaldehyde	9.00	31.35 ± 9.34	14.81 ± 3.00	21.91 ± 3.53	12.23 ± 1.60
(E,E)-2,4-Nonadienal	6.00	–	–	7.43 ± 0.59	–
(E,E)-2,4-Heptadienal	3.50	–	–	3.64 ± 0.85	2.31 ± 1.24
(E,E)-2,4-Decadienal	0.50	–	–	239.03 ± 59.11	11.40 ± 2.49
(E)-2-Nonenal	0.07	186.76 ± 18.10	125.88 ± 26.42	295.63 ± 48.94	289.39 ± 15.62
Heptanal	10.00	13.04 ± 1.99	2.74 ± 0.67	12.50 ± 2.18	13.32 ± 2.60
Decanal	0.90	40.75 ± 4.49	14.86 ± 4.17	119.59 ± 17.99	12.86 ± 3.04
Hexanal	7.50	68.47 ± 35.67	50.69 ± 8.61	130.02 ± 18.13	43.18 ± 5.35
Nonanal	3.50	191.79 ± 59.60	35.16 ± 12.50	340.94 ± 13.54	60.36 ± 10.62
Pentanal	200.00	–	–	1.88 ± 0.96	–
3-methyl-Butanal	200.00	2.28 ± 0.63	–	1.15 ± 0.16	–
Octanal	47.00	11.21 ± 3.58	1.27 ± 0.41	8.83 ± 1.74	4.04 ± 1.67
3-Pentanone	3.00	25.19 ± 2.79	–	4.94 ± 1.50	–
(E)-2-Propenoic acid, 3-phenyl-, methyl ester	1.00	–	–	10.22 ± 4.70	2.19 ± 1.16
Allyl propyl ester	30.00	3.60 ± 1.62	–	–	–
1-Octanol	54.00	1.48 ± 0.44	–	2.44 ± 0.47	3.41 ± 1.58
1-Octen-3-ol	2.00	107.78 ± 28.13	32.48 ± 11.41	313.71 ± 56.70	66.42 ± 1.58
(E)-2-Octen-1-ol	50.00	1.96 ± 0.88	–	3.14 ± 0.72	–
Trimethyl-Pyrazine	10.00	1.13 ± 0.13	–	1.83 ± 0.64	–
P-Cresol	21.00	–	3.79 ± 2.44	3.32 ± 1.72	–

Values are expressed as mean ± standard deviation.

Aldehydes account for a relatively large portion of ham flavor. Fifteen aldehydes were screened from different chewing stages, and aldehydes have unique flavor characteristics. For example, hexanal and heptanal are formed during the oxidation of ham lipids; hexanal has a tallow aroma and is usually the main breakdown product of linoleic acid. Heptanal is a breakdown product of unsaturated fatty acids and exhibits a smoky flavor when heptanal exceeds a threshold. Aldehydes such as heptanal and hexanal are common ham-critical flavor substances detected in all treatment groups.

Volatile flavor compounds such as trans-2,4-heptadienal (fatty and fruity flavor) and trans-2,4-decadienal (chicken fat flavor) were detected in the ultrasonicated group as compared to the non-ultrasonicated group, suggesting that ultrasonication can assist in the production of flavor compounds (38). The types and contents of aldehydes key flavor substances were significantly higher in the UD group than in the other groups, which may be due to the fact that ultrasonic treatment facilitates the release of flavor substances from the ham and optimizes the chewing time of the mouth, so that the contents and types of flavor substances in its bolus were significantly higher than in the other treatment groups.

Alcohols are the main flavor products of fat oxidation in ham. Depending on the degree of oxidation, unsaturated fatty acids are reduced to different fatty alcohols. Unsaturated fatty alcohols have a lower odor threshold and are able to detect more volatile flavor substances (39). The OAV of 1-octen-3-ol, the key flavor substance of alcohols, is higher than that of other alcohol compounds and plays an important role in ham flavor.

Ketones, esters, pyrazines, and phenolic compounds screened for key volatile flavor compounds at different chewing stages, and these compounds also play an important role in flavor perception at different chewing stages. Combined with Table 3, the analysis of Figure 3 shows that there are differences in the types and contents of key flavor substances among the four groups CD, CH, UD, and UH. UD and UH had more flavor categories than the CD and CH groups, and volatile flavor compounds were more readily perceived in the UD and UH groups after sonication.

#### 3.3.2 Electronic nose analysis

The electronic nose is sensitive to odor, and the change of the electronic nose is reflected in its sensor response. Electronic nose technology is an important means of analyzing the flavor of food, which can be comprehensively analyzed and is widely used in the food industry (40).

**Figure 4:**
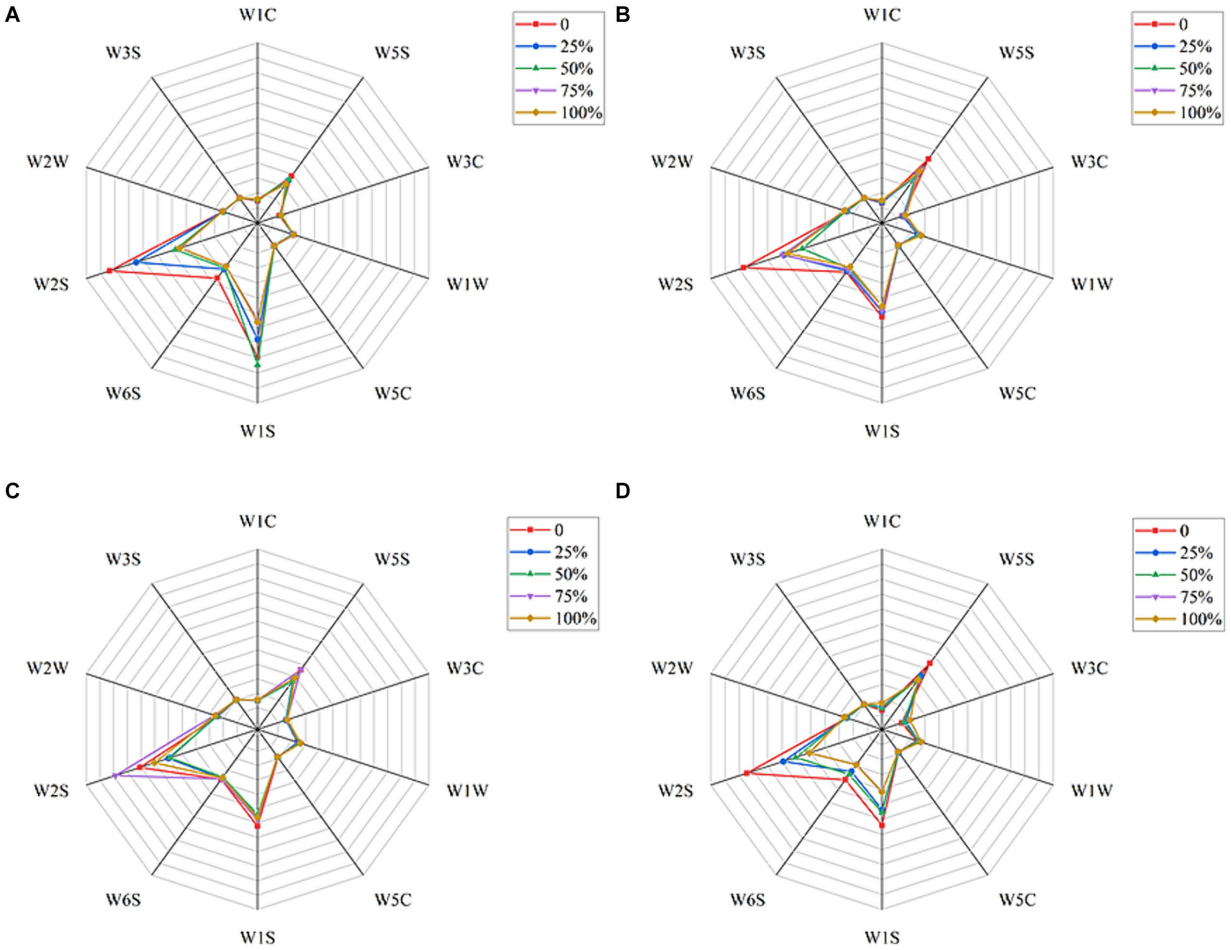
Radargrams for **(A)** CD; **(B)** CH; **(C)** UD; **(D)** UH with different levels of mastication.

The e-nose results for different chewing stages of the bolus are shown in Figure 4. The four most sensitive sensors were W2S, W1S and W5S, indicating that the main flavor compounds were alcohols, aldehydes, ketones, nitrogen oxides and methane compounds. Combined with the joint analysis in Table 3, it can be concluded that the main compounds are aldehydes. From the analysis of Figure 4, it can be seen that the amount of flavor compounds detected in the CD, CH, and UH groups decreased with the increase in the degree of chewing. However, W2S in the UD group was instead most sensitive at the 75% mastication stage, which may be due to the fact that the ultrasound treatment resulted in the longest mastication time in the UD group, which was more sensitive to the W2S sensor at the 75% mastication stage.

**Figure 5:**
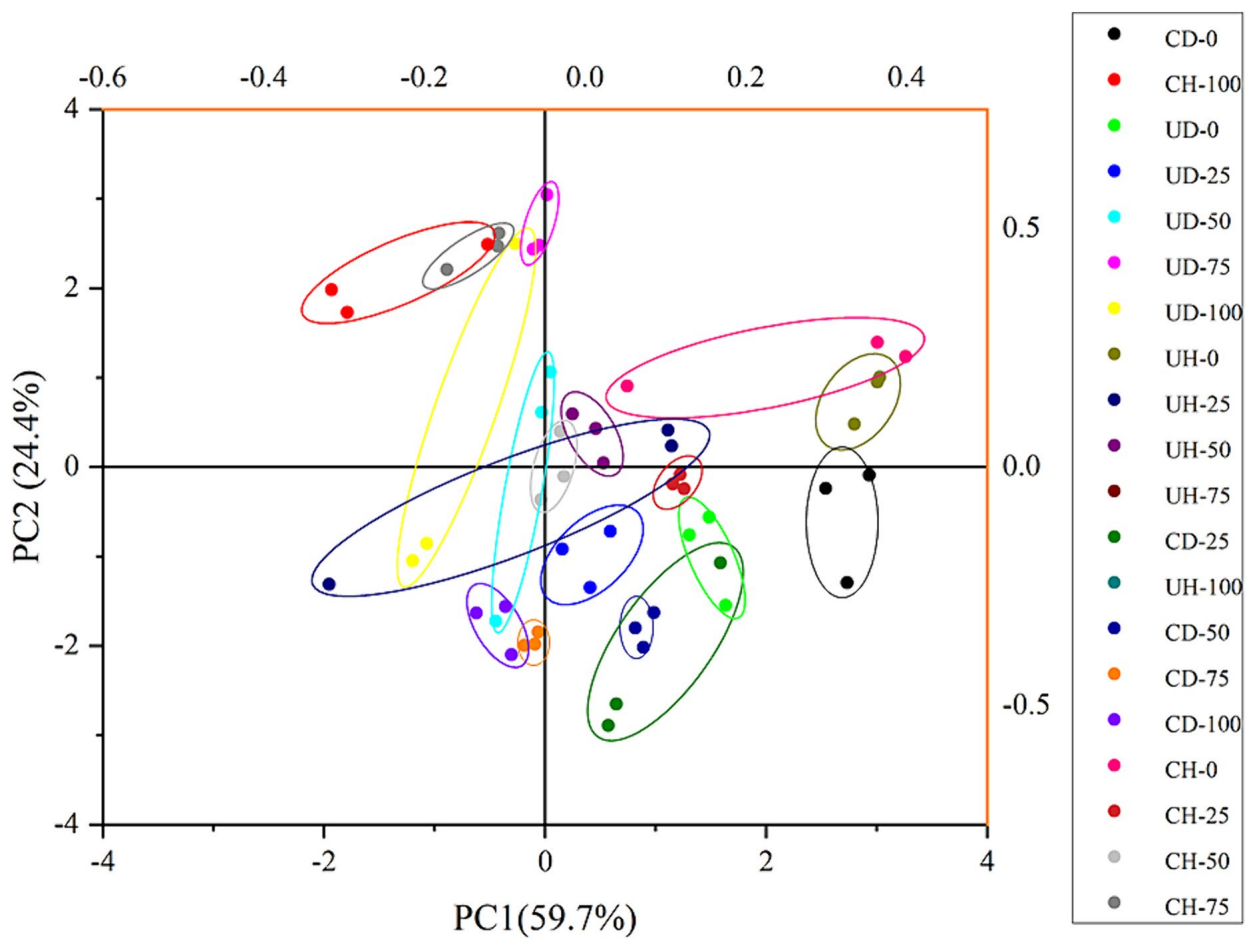
E-nose PCA analysis of boluses with different levels of chewing.

The results obtained from the electronic nose sensors were analyzed using PCA (Figure 5). As depicted in Figure 5, the main components PC1 and PC2 contributed 59.7 and 24.4%, respectively, to the aroma of the four groups of doughs with varying levels of chewing. The cumulative contribution rate of these components reached 84.1%, effectively capturing the differences in aroma components among the four groups of bolus with different chewing degrees. Notably, the UD and UH groups exhibited close proximity in terms of chewing time, indicating a high similarity in overall aroma, as also supported by the analysis in Table 3. On the other hand, samples within the same group but with different chewing times were widely scattered, indicating significant variations in aroma components at different chewing levels. This suggests that aroma perception is influenced by the level of chewing. Furthermore, the volatile compound compositions of the different treatment groups displayed diverse sensitivities to the 10 sensors in the electronic nose, thereby impacting the final results.

### 3.4 TDS analysis

TDS is a sensory evaluation method that requires evaluators to continuously indicate the dominant sensation throughout a given period of time. The “dominant sensation” is defined as the one that captures the most attention over time. TDS allows for the collection of sensory characteristics perceived at different time points during the chewing process. In this study, TDS was applied to analyze flavor changes in four groups of bolus with different chewing levels, namely CD, CH, UD, and UH. Sensory attributes such as ham firmness, saltiness, juiciness, gelatinousness, acidity, and softness were identified as key factors affecting consumer acceptability (41).

**Figure 6:**
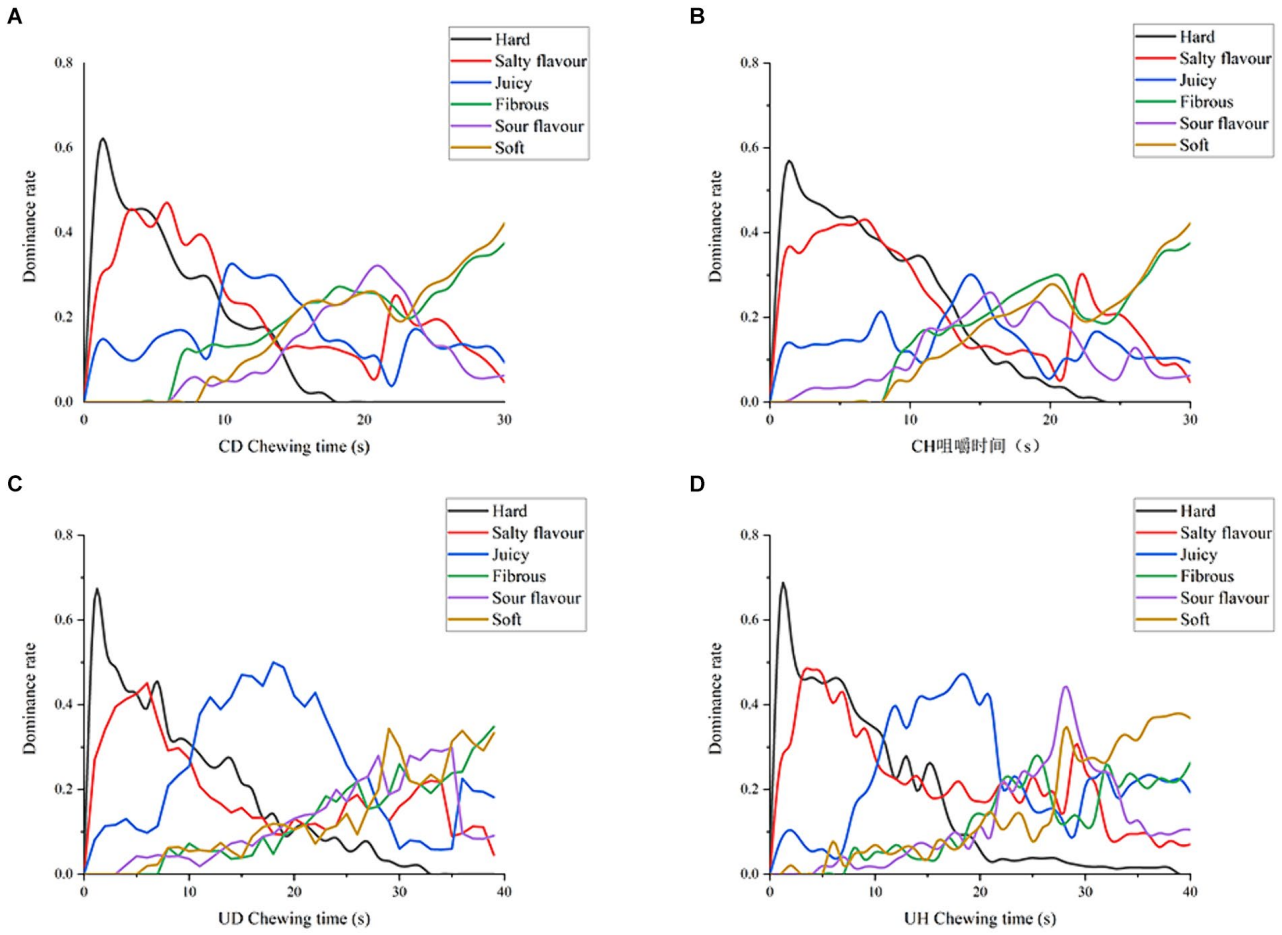
TDS plots of **(A)** CD, **(B)** CH, **(C)** UD, **(D)** UH.

Figure 6 depicts the differences in ham perception across treatment groups for the four groups of hams at the time of first chewing to swallowing. For the CD group (Figure 6A), the sensation within 5 s of chewing was mainly related to hardness, the dominant sensation between 5 s-10s was saltiness, juiciness was the dominant sensory attribute between 10s-15s, sourness was dominant near 20s, and gelling and softness had the highest rate of dominance in the final sensation. In the CH group, firmness dominated the sensory attributes (1–10s), followed by saltiness and hardness together as sensory dominant attributes at 5–10s, juiciness reached its maximum perception at 15 s, and similarly gumminess and softness dominated the final chewing time.

For UD (Figure 6C), the sensory predominance within 10 s of chewing was related to hardness, the sensory predominance within 10–25 s of chewing time was juiciness, the sensory predominance within 25–35 s of chewing was softness, and the sensory predominance attribute of the last 5 s of chewing time was gumminess. Saltiness in UD perception declined continuously after 5 s of chewing, but saltiness perception increased slightly again at 25–35 s of chewing time. Sourness is also perceived to peak around 35 s of chewing time, which may occur when chewing time is about to reach the swallowing point and the flavor is more easily perceived in ham. For UH (Figure 6D), the overall sensory perception was similar to UD. However, the dominance of hardness exhibited from Figure 6D is higher than UD, which is due to the fact that the hardness of the UD sonicated group is lower than that of UH. UH salty and sour taste perception was higher than UD, UH sour taste dominated the senses for 25–30 s, and the overall intensity of salty taste perception was higher than UD. This is because UH has a lower desalination rate than UD, which has a higher degree of protein hydrolysis, resulting in a saltier and more acidic ham sample. Final swallowing phase softness and gelling UD and UH showed similar perceptions because the water content of the bolus was similar when reaching the end point of swallowing, resulting in similar softness and gelling in the TDS images.

## 4 Conclusion

The present study showed that there was no significant difference in flavor perception among the different treatment groups in oral processing. However, the chewing time and chewing frequency of the UD group were significantly higher than those of the other groups, and prolonged chewing would more easily lead to satiety, which in turn would reduce the amount of food eaten to play a role in weight loss. The analysis of GC–MS and electronic nose allowed us to conclude that the ultrasonicated hams produced more types and amounts of key flavor substances during chewing than the non-ultrasonicated group. The UD group had more critical flavors than the other groups, which suggests that the UD group is more capable of producing flavor substances during chewing, which are more acceptable to consumers. From the TDS, it can be seen that all treatment groups did not differ significantly in dominant sensory attributes, but UD was less dominant in sensory attributes such as saltiness and sourness, which are not accepted by consumers and are more likely to be accepted by consumers. Overall analysis UD flavor is more easily perceived during consumer chewing. This study is the first to analyze the oral processing characteristics of ultrasonically treated hams, and to represent the perception of ham texture and flavor at different chewing stages by analyzing bolus with different levels of chewing.

## Data Availability

The original contributions presented in the study are included in the article/supplementary material, further inquiries can be directed to the corresponding author.
